# Development and Challenges of Food Contaminant Removal Technologies: Molecular Imprinting Technology as an Emerging Solution

**DOI:** 10.3390/nano16150954

**Published:** 2026-08-03

**Authors:** Qian Guo, Yawei Xiong, Jing Neng

**Affiliations:** College of Food Science and Engineering, Zhejiang University of Technology, Deqing 313299, China; 201806022508@zjut.edu.cn (Q.G.); 211124260012@zjut.edu.cn (Y.X.)

**Keywords:** molecularly imprinted polymers (MIPs), food contaminant removal, pesticide residues, heavy metals, biotoxins, plasticizers, selective adsorption

## Abstract

Food contaminants, including plasticizers, pesticide residues, heavy metals, and biotoxins, pose persistent risks to food quality and human health. Their diverse sources, complex migration pathways, and potential long-term toxicity make removal difficult. Conventional removal technologies, such as physical treatment, chemical degradation, adsorption, membrane separation, and biological methods, can reduce contaminant levels to varying degrees. However, they often show limited selectivity, matrix interference, harsh operating requirements, or losses of nutritional and functional components. Molecularly imprinted polymers (MIPs) are synthetic recognition materials with binding sites tailored to a target contaminant. Their template-induced cavities provide complementarity in size, shape, and functional-group arrangement, enabling selective adsorption in complex matrices. Recent studies apply MIPs to the enrichment, detection, and removal of plasticizers, pesticide residues, heavy metals, and biotoxins. Unlike recent surveys centered on MIP-assisted analysis and sensing, this review uses contaminant removal as the organizing problem and compares MIP-based strategies with conventional decontamination across four hazard classes. MIPs offer tunable selectivity, chemical stability, and reusability, but practical food applications still face template leakage, slow mass transfer, incomplete safety evaluation, matrix dependence, and scale-up limitations. Future work should prioritize green synthesis, surface imprinting, magnetic recovery, and systematic validation in real food matrices. To prevent analytical extraction from being conflated with remediation, the evidence is classified from proof-of-binding and analytical cleanup to edible-matrix treatment and process validation, and representative studies are compared using capacity, removal or recovery, equilibration time, selectivity, reuse, and matrix validation. Recent evidence also reveals substantial gaps for PFAS, microplastics, and nanoplastics: selective recognition is advancing, but food-safe removal remains largely unvalidated.

## 1. Introduction

In recent years, food safety has attracted increasing attention from both the public and scientific communities. Among various food contaminants, plasticizers, pesticide residues, heavy metals, and biotoxins have become major factors affecting food quality and human health due to their diverse sources, high concealment, and significant toxicity. Although conventional physical, chemical, and biological removal technologies can reduce contaminant levels in foods to some extent, they still commonly suffer from several limitations, including insufficient selectivity, harsh processing conditions, potential adverse effects on nutritional and sensory quality, and poor adaptability to complex food matrices. Therefore, the development of efficient, green, highly selective, and food-compatible technologies for contaminant removal is of great significance.

Molecularly imprinted polymers (MIPs) contain template-directed recognition sites and combine chemical stability with reusable target binding. These properties support selective adsorption, separation, and analytical cleanup of food contaminants. This review evaluates current food contaminant removal technologies and summarizes the principles, preparation strategies, and applications of MIPs for plasticizers, pesticide residues, heavy metals, and biotoxins. It also examines the conditions that determine whether selective binding can translate into safe removal from real food matrices.

Several recent reviews have examined MIPs in food science from complementary perspectives. Existing syntheses address broad food applications [1,2] and food processing or quality control [3]. Other reviews focus on optical or multimodal contaminant sensors [4,5], surface-enhanced Raman spectroscopy (SERS)-assisted trace analysis [6], emerging-pollutant analysis [7], or multi-analyte and green-synthesis strategies [8]. Importantly, prior perspectives have already recognized extraction and possible food decontamination as MIP applications [3,9]. The novelty of the present review is therefore not a claim to be the first review of removal. Instead, it addresses a methodological gap by separating analytical cleanup of a small aliquot from treatment of a bulk edible matrix; linking removal performance to binding chemistry, site heterogeneity, and mass-transfer architecture; and benchmarking MIPs against conventional decontamination using matrix tolerance, food-quality retention, sorbent recovery, leakage, regeneration, throughput, and regulatory readiness. This evidence-centered framework tests whether imprint-derived selectivity survives competitive adsorption and transport limitations in real foods.

## 2. Overview of Molecular Imprinting

### 2.1. Synthesis of MIPs

There are various methods for preparing MIPs, and different preparation strategies can endow the resulting materials with distinct host–guest polymer characteristics. These methods mainly include: (1) monomer polymerization in the presence of template molecules; (2) phase inversion induced by incompatible solvents or solvent evaporation; and (3) surface imprinting based on soft lithography [10,11]. In general, the preparation of MIPs involves mixing functional monomers with template molecules, carrying out polymerization around the template molecules in the presence of crosslinkers, and finally removing the template molecules to form polymers with specific binding sites [10,12,13].

**Figure 1:**
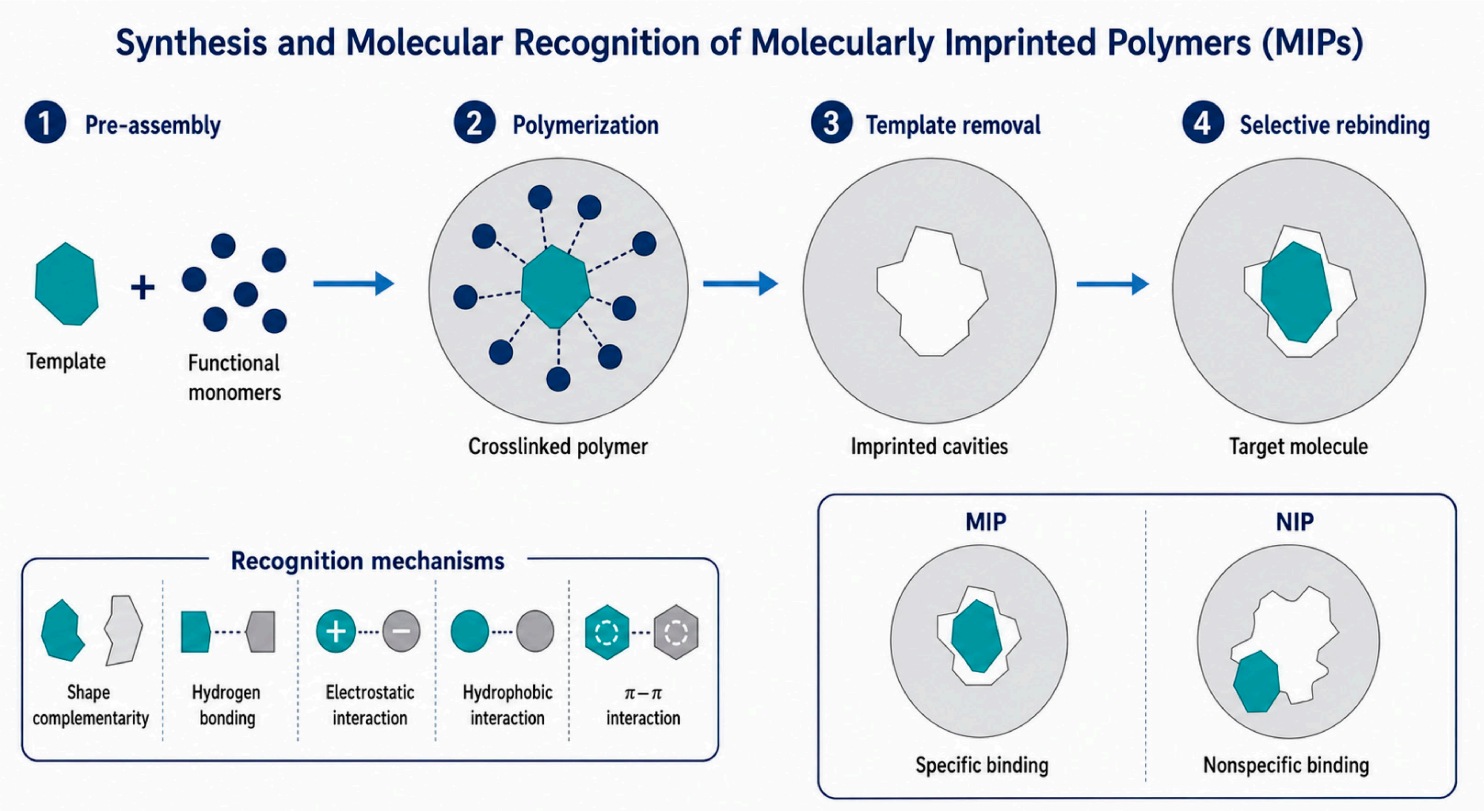
Synthesis process and molecular recognition mechanism of MIPs.

As shown in Figure 1, molecular imprinting is achieved by introducing crosslinkers around functional monomers and template molecules during polymerization. First, the template molecules interact with complementary functional monomers to form template–monomer complexes, and the specific mode of complex formation is a key factor distinguishing different molecular imprinting techniques. Subsequently, crosslinking polymerization is carried out around the complexes. After template extraction, the resulting imprinted sites possess a three-dimensional network structure, in which the pore geometry and spatial distribution of functional groups are highly complementary to the functional groups and spatial configuration of the template molecules [10,12,14]. The preparation of MIPs generally includes template–functional monomer preassembly, crosslinking polymerization, template elution, and target molecule re-recognition. During polymerization, the template molecules induce the formation of recognition sites with specific spatial structures and functional group distributions. After elution, cavities matching the size, shape, and interaction sites of the target molecules are retained within the polymer, thereby enabling selective recognition and adsorption of target contaminants in subsequent applications.

Taking free-radical and electropolymerized systems as examples, MIP synthesis relies on covalent or non-covalent interactions between functional monomers and template molecules. Common vinyl and acrylic monomers include methacrylic acid, acrylic acid, acrylamide, 2- or 4-vinylpyridine, methyl methacrylate, 2-hydroxyethyl methacrylate, and N-isopropylacrylamide. Electropolymerized MIPs frequently use aniline, pyrrole, o-phenylenediamine, dopamine, or aminophenylboronic acid. Sol–gel systems often employ silane monomers such as 3-aminopropyltriethoxysilane (APTES). These examples are not exhaustive because monomer selection depends on template acidity, basicity, hydrogen-bonding and coordination sites, hydrophobicity, and polymerization medium [15]. After crosslinkers such as ethylene glycol dimethacrylate (EGDMA) and initiators such as azobisisobutyronitrile (AIBN) are added, polymerization can be initiated by heat or ultraviolet irradiation. Phase inversion instead induces polymer precipitation with an incompatible solvent or forms a thin film by solvent evaporation. Soft lithography presses a stamp prepared from a template array into a partially polymerized film. After polymerization, stamp removal and template washing generate surface-accessible binding sites [10,12,14].

The synthesis of MIPs involves several key components, including template molecules, functional monomers, crosslinkers, polymerization initiators, and solvents, and the selection of these components directly affects the performance of MIPs [16]. The template molecule is the core element in MIP synthesis. An ideal template should contain functional groups that do not interfere with the polymerization reaction, exhibit strong chemical stability, and be capable of forming complexes with functional monomers [17]. In recent years, metal ion imprinting technology has improved selectivity and adsorption efficiency by using metal ion–ligand complexes as templates, thereby overcoming the limited selectivity of conventional metal ion imprinting approaches [18,19].

Functional monomers form pre-polymerization complexes with template molecules through specific functional groups and are therefore critical for determining the recognition performance of MIPs. In the design of functional monomers, the recognition units and polymerizable moieties, such as vinyl or silanol groups, should act synergistically to enhance the binding affinity toward template molecules [20]. In addition, fragment imprinting technology employs partial structural units of target molecules as pseudo-templates, which not only improves recognition capability but also broadens the application scope of MIPs in trace analysis and separation from complex matrices [21].

Crosslinkers fix the spatial positions of functional monomers during polymerization, thereby forming stable three-dimensional network structures. The type and dosage of crosslinkers have significant effects on the mechanical properties and recognition ability of MIPs. Studies have shown that novel crosslinkers, such as rosin derivatives, can not only increase the degree of crosslinking but also serve as ideal materials owing to their renewability and low toxicity [22]. Porogens determine the morphology and adsorption performance of the polymer. Non-polar and weakly polar solvents, such as chloroform and acetonitrile, are commonly used in non-covalent imprinting to improve imprinting efficiency [23]. Room-temperature ionic liquids (RTILs), as emerging porogens, can reduce solvent volatility and significantly enhance imprinting efficiency and adsorption selectivity [24]. Initiators are usually azo compounds, such as AIBN, and are mainly used in free-radical polymerization [25]. During polymerization, dissolved oxygen is often removed using inert gases, such as nitrogen, to avoid oxygen-induced inhibition. In addition, photoinitiators and other alternative initiation techniques have gradually been explored to meet the requirements of specific applications [26].

Overall, the rational selection and optimization of template molecules, functional monomers, crosslinkers, solvents, and initiators, together with improvements in polymerization processes, not only determine the selectivity, adsorption capacity, and stability of MIPs, but also promote their broad application in complex sample pretreatment, selective separation, and detection.

When selecting a preparation method for MIPs, the specific requirements of the intended application should be comprehensively considered, although some preparation processes remain relatively complex. Regardless of the preparation method used, the morphology of the resulting materials is typically characterized by electron microscopy or atomic force microscopy to analyze their pore structures and evaluate their potential in applications such as sensing [27,28].

### 2.2. Broad Applications of MIPs in Adsorption and Separation

MIPs are polymeric materials whose spatial structures and binding sites are highly complementary to the corresponding template molecules. After removal of the template molecules, three-dimensional cavities matching the size and shape of the templates are retained within the polymer network. These cavities contain binding sites capable of recognizing the template molecules, thereby endowing MIPs with highly selective recognition performance and strong specificity toward target substances to be separated [28]. Since Wulff et al. [29] first successfully prepared MIPs in 1972, molecular imprinting technology has developed rapidly. In particular, since 2000, a large number of application patents related to molecular imprinting technology have emerged worldwide and have shown an increasing trend year by year.

**Figure 2:**
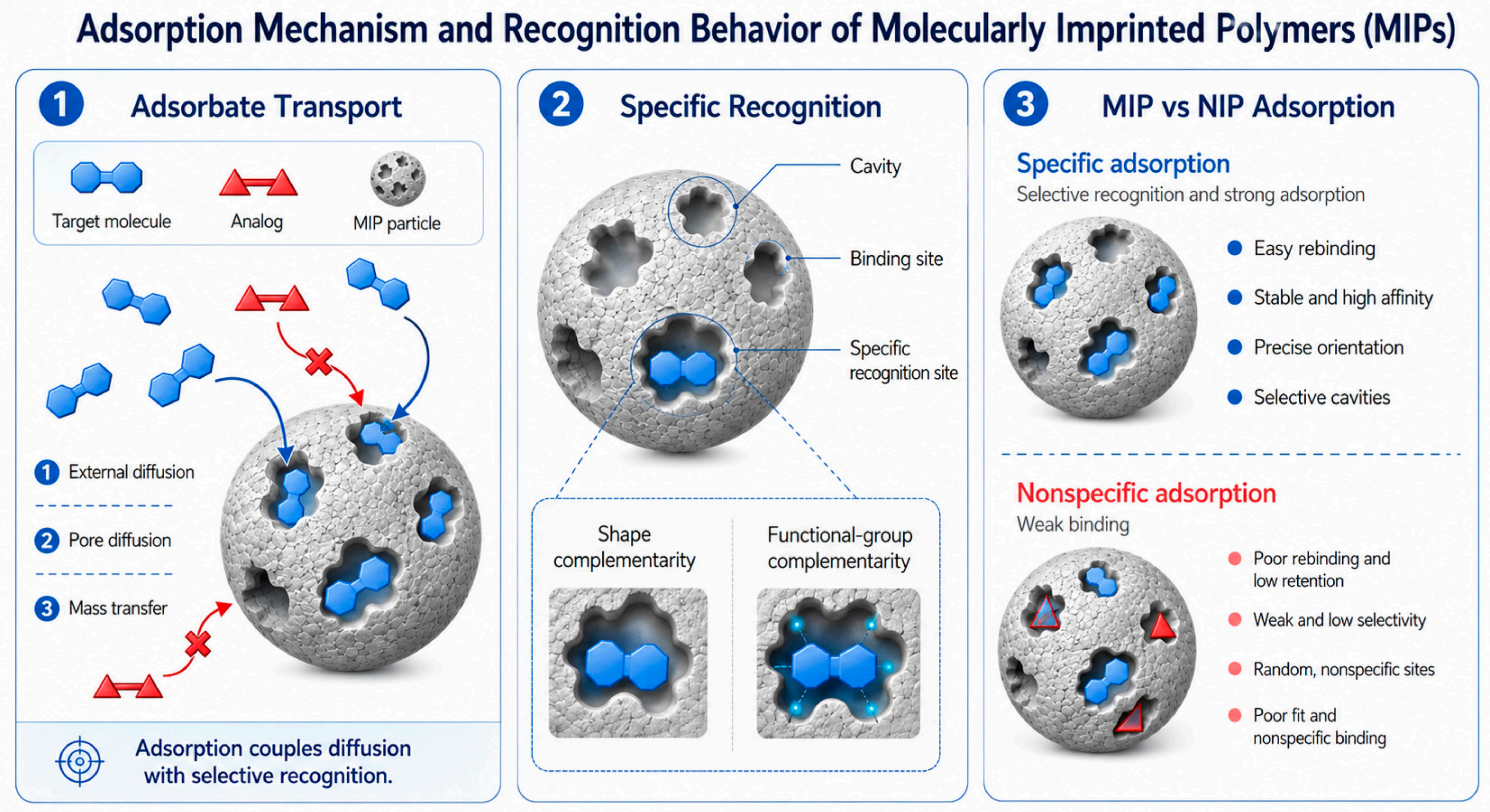
Adsorption mechanisms and molecular recognition behavior of MIPs.

Owing to their excellent physicochemical properties and specific recognition ability toward template molecules, MIPs have been widely applied in solid-phase extraction, chiral separation, trace analysis, biomimetic sensors, catalysis, and selective adsorption [6]. As shown in Figure 2, adsorption does not simply depend on pore area. Complementary cavity geometry and the spatial arrangement of functional groups promote hydrogen bonding, ionic or coordination interactions, hydrophobic partitioning, π–π interactions, and van der Waals forces. Geometric matching alone, however, is not an adsorption mechanism. The target must cross an external liquid film, partition into the pore liquid or swollen polymer, diffuse to an accessible site, and then associate with imprinted or non-imprinted regions.

The observed rate and capacity are therefore jointly controlled by particle size, pore connectivity, imprinted-layer thickness, crosslink density, polymer swelling, mixing, food viscosity, and the target distribution among aqueous, lipid, protein-bound, and particle-bound phases. Water can weaken hydrogen bonding, salts screen electrostatic attraction, and endogenous chelators alter metal-ion speciation. Hydrophobic and aromatic surfaces may improve uptake of phthalates, pesticides, or toxins from water but also attract lipids, pigments, and polyphenols. Surface imprinting shortens diffusion paths but can expose more nonspecific area; magnetic cores improve recovery but do not intrinsically increase affinity. Thus, architecture changes both recognition and engineering trade-offs [30,31].

At equilibrium, uptake should be reported as *q_e_* = (*C*_0_ − *C_e_*)*V*/*m*, together with the concentration range, phase ratio, and uncertainty. Langmuir, Freundlich, Sips, and dual-site models describe data but do not identify a binding force. The homogeneous-site assumption of Langmuir is often questionable for non-covalent MIPs, whereas Freundlich has no finite saturation capacity. Likewise, a superior pseudo-second-order fit is not proof of chemisorption; nonlinear fitting, residuals, confidence intervals, and transport experiments are needed [32,33]. Selectivity should be measured under matched competitive conditions using a defined imprinting factor (IF = q_MIP_/q_NIP_), distribution coefficients, target-to-competitor selectivity, and relative selectivity against the NIP. For treatment claims, dynamic capacity, breakthrough, mass-transfer-zone behavior, mass balance, and regeneration are more process-relevant than a batch fit alone.

Commercialization has not progressed linearly, and static company lists repeated from early literature no longer provide a reliable picture of current activity. Recent commercialization reviews indicate that market offerings remain limited and concentrate on custom MIP synthesis, solid-phase extraction sorbents, affinity reagents, and sensing components [34,35]. Commercial availability also does not establish food-grade approval or readiness for direct food processing. For contaminant removal, the main translation gaps are batch reproducibility, scalable manufacture, material recovery, regulatory evaluation, and validation in real food matrices.

For example, Lv et al. [36] prepared an imprinted polymer using the insecticide dimethoate as the template molecule and found that the recovery of spiked dimethoate from the imprinted polymer column reached 100%. Zhao et al. [37] prepared an MIP sensor using glyphosate as the template molecule and found that the sensor exhibited high selectivity toward glyphosate even in the presence of interfering matrix components. Zhao et al. [38] from Inner Mongolia University of Science and Technology prepared MIPs for the adsorption and separation of plasticizers using butyl benzyl phthalate as the template. The experimental results showed that the adsorption equilibrium time of the imprinted polymer for the plasticizer was 5 h, and that the polymer exhibited good adsorption performance and excellent adsorption selectivity toward plasticizer molecules.

Boontongto et al. [39] and Seebunrueng et al. [40] developed magnetic MIPs for the selective extraction of organophosphorus pesticides from fruit and vegetable samples. These studies demonstrate effective molecular recognition and matrix clean-up, but their analytical recoveries and detection performance should not be interpreted as evidence of contaminant removal from bulk foods.

Compared with conventional adsorbents such as silica gel, molecular sieves, activated carbon, diatomite, aluminum oxide, and metal–organic frameworks, MIPs combine target-directed binding with good thermal and mechanical stability. Many monomers and crosslinkers are bulk chemicals, and suspension or emulsion polymerization can support production at larger scale. However, low-cost raw materials do not by themselves establish industrial feasibility. Batch reproducibility, solvent use, template removal, particle recovery, regeneration, and food-grade safety remain decisive constraints [1,34].

## 3. Sources, Hazards, and Removal Challenges of Major Food Contaminants

With social development, consumers have placed increasingly high demands on food quality and safety. Meanwhile, the diversification of food products, the expansion of food production and acquisition modes, such as cultured meat and genetically modified foods, and the emergence of increasingly advanced food detection technologies have brought more food safety issues into public view. Food contaminants are among the major causes of food safety problems. In the investigation conducted by Koch et al. [41], undesirable substances present in many foods were shown to pose serious food safety and health risks. However, the contaminants that may occur in foods are highly diverse, including environmental pollutants, processing-induced contaminants, unauthorized adulterants and food additives, migrants from packaging materials, and naturally occurring contaminants. These contaminants can be further classified into pesticides, plasticizers, heavy metals, biotoxins, and other categories [42]. Such hazardous substances in foods pose substantial threats to human health. Food contaminants originate from complex sources, including migrants from packaging materials, pesticide residues, heavy metals, biotoxins, and emerging contaminants that have attracted increasing attention in recent years [43].

**Figure 3:**
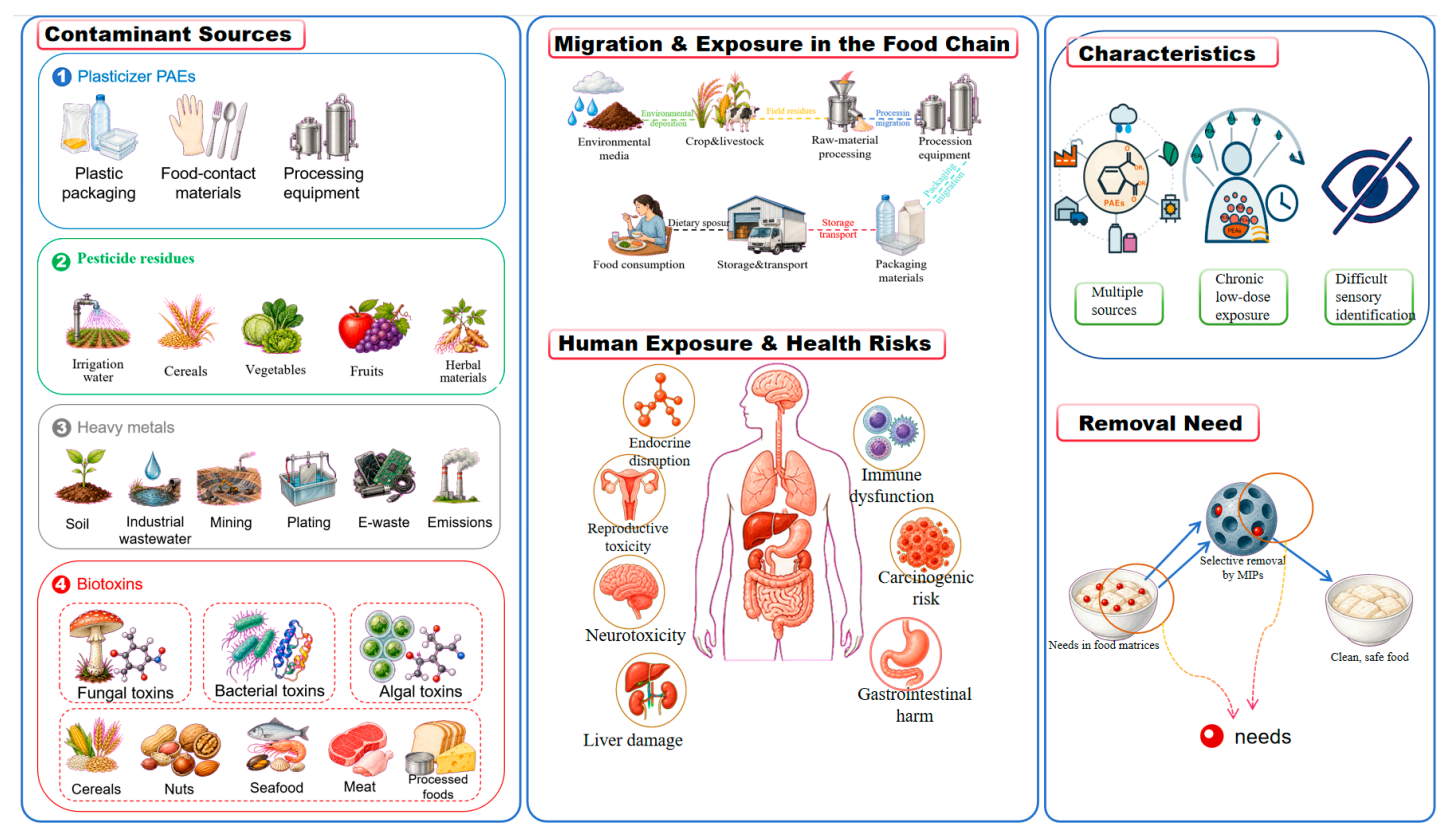
Major sources of food contaminants, their migration and exposure pathways throughout the food chain, associated human health risks, and the resulting need for selective contaminant-removal technologies. MIP, molecularly imprinted polymer.

As shown in Figure 3, food contaminants have complex sources, including migration from packaging materials, pesticide residues from agricultural production, heavy metal contamination from environmental media, and biotoxins produced by molds, bacteria, or algae. These contaminants may migrate or accumulate during food production, processing, storage, transportation, and consumption and subsequently enter the human body through the food chain. They may further induce endocrine disruption, neurotoxicity, liver and kidney damage, immunosuppression, and potential carcinogenic risks. Therefore, the development of efficient, mild, and selective removal technologies is an important requirement for ensuring food quality and safety.

Plasticizers, as commonly used plastic additives, can impart excellent flexibility and processability to plastics. Their global annual production exceeds 8 million tons, with China being the largest producer and user, producing approximately 4.3 million tons annually. Phthalate esters (PAEs) are the most widely used plasticizers, accounting for more than 80% of total plasticizer consumption [44], and are extensively applied in plastics, cosmetics, pharmaceuticals, packaging materials, and construction industries. Plasticizers can migrate over time into air, soil, and water, and eventually enter living organisms. This issue is particularly prominent during food processing and storage. When plastic packaging materials or processing equipment are used under high-temperature or lipid-rich conditions, PAEs are prone to migration into foods. In recent years, several food safety incidents involving plasticizers have raised public concern regarding their potential health risks [45].

Pesticides are chemical agents, biological preparations, or mixtures used to control pests, plant diseases, and weeds, and are widely applied in agriculture and public health management [46,47]. In some cases, the use of inferior raw materials, illegal additions during production, and irregular circulation during sales have contributed to pesticide residues becoming an important global issue in food safety and public health [36]. Pesticides can enter the human body through grains, vegetables, fruits, Chinese medicinal materials, drinking water, feed, and other routes. Long-term exposure may cause damage to the nervous, hematological, and skeletal systems, as well as carcinogenic, teratogenic, and mutagenic risks. Pesticides may also cause acute poisoning, with children being more sensitive to their toxic effects [48].

The sources of plasticizers and pesticide residues in foods are closely associated with multiple stages of the food industry chain, including excessive pesticide application during agricultural cultivation, harvesting before the required safety interval, release from packaging materials and processing equipment, illegal use during food additive processing, and contamination from environmental media [49]. In addition, insufficient regulatory systems and limited detection capacity further aggravate these risks. Improper storage practices by consumers may also lead to additional contamination. The combined effects of these factors make plasticizers and pesticides long-standing hazards in the field of food safety and have promoted research on technologies for their removal [50,51,52].

Heavy metal contamination is also a major concern in food safety. Common heavy metals include lead, cadmium, mercury, and arsenic, which may originate from soil, groundwater, mineral resources, industrial wastewater, agricultural fertilizers, and environmental emissions [53]. Heavy metals can accumulate in animals and plants and be transferred to humans through the food chain. After entering the human body, they may accumulate in organs such as the liver, kidneys, and brain, interfere with enzymatic activity and multiple physiological systems, and cause serious health consequences, including immune dysfunction, neurodevelopmental disorders, kidney injury, and even cancer [54].

Biotoxins are mainly produced by molds, bacteria, and algae, and include aflatoxins, marine toxins, and bacterial exotoxins [55]. They may be present in cereals, nuts, seafood, meat products, and improperly stored foods. Due to their strong toxicity and thermal stability, biotoxins are difficult to completely eliminate by conventional heating. Once ingested, they may induce severe adverse effects, such as liver injury, immunosuppression, neurological symptoms, and acute gastroenteritis [56,57]. These contaminants are often difficult to identify by sensory evaluation and are highly concealed, thereby posing persistent threats to food safety and public health.

As shown in Table 1, in addition to conventional food contaminants, emerging contaminants such as per- and polyfluoroalkyl substances (PFAS), microplastics, nanoplastics, and engineered nanoparticles have also received increasing attention. These contaminants are generally characterized by strong environmental persistence, complex sources, diverse migration pathways, and high detection difficulty. They can enter food systems through food contact materials, processing equipment, environmental media, and bioaccumulation along the food chain. Compared with conventional contaminants, the toxicological mechanisms, exposure levels, limit standards, and removal methods for emerging contaminants remain insufficiently established. Moreover, they may form combined contamination with other organic pollutants or metal ions. Therefore, the development of highly selective and food-compatible removal materials for emerging contaminants represents an important direction for future food safety research.

## 4. Conventional Contaminant Removal Technologies and Their Applications

The selection of food contaminant removal technologies is usually closely related to the type of contaminant, characteristics of the food matrix, processing conditions, and safety requirements. At present, conventional methods for removing contaminants such as plasticizers, pesticide residues, heavy metals, and biotoxins from foods mainly include physical treatment, adsorption separation, chemical degradation, biodegradation, membrane separation, and supercritical extraction. These methods have been applied to some extent in food processing, sample pretreatment, and contaminant control and have played a positive role in reducing contaminant levels and improving food safety. Among them, physical treatment methods are generally simple to operate and relatively low in cost; adsorption separation technologies have a wide application range and can be readily integrated into existing processing procedures; chemical degradation and advanced oxidation technologies exhibit high treatment efficiency for certain refractory contaminants; biological methods are mild and environmentally friendly; and membrane separation and supercritical extraction technologies show favorable separation efficiency and selectivity in specific systems.

However, food systems are often characterized by complex compositions, abundant nutrients, strong matrix interference, and high quality requirements. Therefore, conventional removal technologies still have certain limitations in practical applications. For example, physical treatment methods have limited ability to remove contaminants located in deep layers or bound states. Although conventional adsorbents are relatively inexpensive, they usually lack selectivity and may simultaneously adsorb bioactive or nutritional components from foods. Chemical degradation methods are efficient, but the formation of by-products and changes in food quality must be carefully considered. Technologies such as membrane separation and supercritical extraction require specialized equipment and strict operating conditions, resulting in relatively high costs for industrial application. Therefore, it is necessary to systematically summarize the advantages and limitations of different conventional removal technologies in the treatment of typical food contaminants, thereby providing a theoretical basis for introducing MIPs with specific recognition capability.

### 4.1. Conventional Methods for Removing Pesticide Residues from Foods

Yang [67] established a method for the determination of multiple pesticide residues in dandelion, honeysuckle, ginseng, and wolfberry using QuEChERS pretreatment combined with ultra-performance liquid chromatography–tandem mass spectrometry (UPLC-MS/MS). The effects of different processing methods, including heating, washing, processing, and light exposure, on the removal of pesticide residues from wolfberry were further investigated. The results showed that different processing methods had significant effects on pesticide removal efficiency. Chafiqi et al. [68] investigated the removal of pesticide residues from lettuce by ultrasonic washing and confirmed that ultrasonic treatment could improve the removal efficiency of pesticide residues to a certain extent. Wu [69] studied the detection and processing-transfer behavior of pesticide residues in root-type Chinese medicinal materials, and the findings were similar to those reported by Sun et al. [70]. In addition, a relatively high pesticide removal rate was achieved using supercritical carbon dioxide extraction. Yang et al. [71] employed a combined ultrasonic ozonation process to remove several common pesticide residues from vegetables, demonstrating promising application potential.

Overall, washing, heating, processing, ultrasonic treatment, supercritical carbon dioxide extraction, macroporous resin adsorption, and advanced oxidation technologies all have certain application value in the control of pesticide residues. Among these methods, traditional processing approaches such as washing, heating, and processing are simple to operate and low in cost, making them suitable for daily processing and preliminary residue reduction. Ultrasonic treatment can enhance mass transfer and improve the removal efficiency of surface pesticide residues. Supercritical carbon dioxide extraction has the advantages of low solvent residue and relatively good selectivity. Macroporous resin adsorption and advanced oxidation technologies also show favorable treatment performance for the removal of specific pesticide residues. However, the application scope and mechanisms of different methods vary, and their removal efficiency is easily affected by pesticide type, residue location, food matrix, and processing conditions. Physical treatments mainly act on the food surface and have limited effects on pesticide residues located in deep layers or strongly adsorbed states. Although supercritical extraction and advanced oxidation technologies are highly efficient, equipment cost, operating conditions, and the potential loss of bioactive components still need to be considered. Adsorption-based methods may be affected by matrix components and still face problems such as insufficient selectivity and unstable regeneration performance. Therefore, in practical applications, conventional pesticide residue removal technologies still require a balance among removal efficiency, food quality preservation, and process feasibility.

### 4.2. Conventional Methods for Removing Plasticizers from Foods

Makzoom et al. [72] used cellulose nanofibers (CNFs) derived from softwood as adsorbents to investigate the removal of di(2-ethylhexyl) phthalate (DEHP) from aqueous solutions. The results showed that CNFs had a certain adsorption capacity for DEHP, and the adsorption performance was affected by factors such as initial concentration, adsorbent dosage, contact time, and pH. This method exhibited the advantages of low cost, abundant raw material sources, and good application potential. However, several limitations remain, including a relatively long adsorption time, material aggregation at high dosages, limited adsorption capacity for high-concentration DEHP, and a complicated regeneration process. Zhang et al. [73] constructed a cutinase-loaded nanoporous gold–polyethyleneimine (NPG-PEI) material for removing DEHP from water. The material showed good removal efficiency and reusability, indicating that the combination of functionalized nanomaterials and enzyme-catalytic systems can improve the treatment efficiency of plasticizer contaminants. Nevertheless, its preparation process is relatively complicated and costly, and its selectivity and applicability in complex food matrices require further verification.

Cellulose-based adsorbents, functionalized nanomaterials, and enzyme-loaded composite materials have certain advantages in plasticizer removal, including abundant raw material sources, favorable adsorption efficiency, good reusability, and enhanced removal capacity toward target contaminants through functional modification. However, current studies are mainly focused on aqueous phases or simulated contaminated systems, whereas application verification in real foods remains relatively insufficient. In particular, for complex matrices that tend to accumulate phthalate plasticizers, such as edible oils and dairy products, conventional adsorbents are easily interfered with by lipids, proteins, and other coexisting components. These materials still face challenges such as insufficient selectivity, limited mass transfer, complicated regeneration, and high cost. Therefore, future plasticizer removal technologies should be further optimized in terms of removal efficiency, selectivity, compatibility with food matrices, and material reusability.

### 4.3. Conventional Methods for Removing Heavy Metals from Foods

Yang et al. [74] prepared amino- and thiol-functionalized materials using dendritic mesoporous silica nanoparticles as carriers and applied them to the adsorption of heavy metal ions such as Cu^2+^, Pb^2+^, and Cd^2+^. The results showed that these materials exhibited good adsorption capacity, selectivity, and cyclic regeneration performance, indicating their potential for heavy metal wastewater treatment. However, such nano-adsorbents are more suitable for environmental water treatment. In complex food matrices, nonspecific adsorption of proteins, lipids, and other components may affect food quality, and issues related to material safety, residual risk, and solid–liquid separation require further evaluation. Zhao et al. [75] pointed out that magnetic solid-phase extraction (MSPE) can achieve rapid enrichment and separation of heavy metal ions using magnetic nano-adsorbents. This method has the advantages of simple operation, high separation efficiency, and low reagent consumption, and has become an important pretreatment technique for the detection of heavy metals in foods. However, MSPE is mainly used for enrichment and purification during laboratory analysis and cannot be directly regarded as a contaminant removal method in food processing. In addition, it still faces challenges in solid or high-viscosity food systems, including dispersibility, material residue, and scale-up application. Xu et al. [76] used a mixed-acid method to remove Pb and Cd from rice residue protein. The results showed that organic acid chelation could effectively reduce heavy metal contents while maintaining the protein structure and processing properties to some extent. This method operates under relatively mild conditions and has certain potential for industrial application, but problems such as high acid consumption, long treatment time, limited removal efficiency for certain metals, and possible effects on flavor and sensory quality remain.

Existing heavy metal removal methods show certain effectiveness in specific systems. Functionalized adsorbents, magnetic solid-phase extraction, and mixed-acid treatment each have their own advantages in heavy metal contamination control. Functionalized porous materials exhibit strong adsorption capacity, magnetic materials facilitate rapid separation, and mixed-acid treatment is relatively mild and suitable for certain food raw materials. However, their practical application in foods is still limited by factors such as material safety, selectivity, compatibility with food matrices, treatment efficiency, and quality preservation. Therefore, the development of heavy metal removal materials that are milder, safer, easily separable, and capable of target recognition remains of great significance.

### 4.4. Conventional Methods for Removing Biotoxins from Foods

Ding [77] investigated the reduction in tetrodotoxin (TTX) using alkaline chemical treatment and microbial fermentation. The results showed that both edible baking soda treatment and *Lactobacillus rhamnosus* fermentation could reduce TTX content to a certain extent. Among them, the microbial method showed a higher toxin reduction effect, whereas alkaline treatment had certain advantages in preserving nutritional components. The author also discussed related strategies in another review [78]. Physical methods, such as heating, temporary rearing, irradiation, and activated carbon adsorption, are relatively simple to operate and convenient for use in some food processing scenarios, but their detoxification effects are limited. Chemical methods, such as weak alkali treatment, ozone oxidation, and photocatalysis, can improve toxin degradation efficiency, but may affect food nutrition and flavor. Biological methods rely on microorganisms or enzymes to degrade toxins and are characterized by mild conditions and good compatibility with food systems; however, their mechanisms of action, treatment stability, and scale-up application still need to be further clarified.

Conventional biotoxin removal technologies have certain application value in specific food systems, and different methods can be selected according to toxin type, food matrix, and processing conditions. However, biotoxins usually have stable structures, strong toxicity, low concentrations, and complex matrix interference, making it difficult for conventional methods to simultaneously achieve high efficiency, safety, selectivity, and food quality preservation. Physical methods generally have limited detoxification effects, chemical methods may affect food nutrition and flavor, and the mechanisms, processing stability, and large-scale application of biological methods remain to be further investigated. Therefore, the development of functional materials with specific recognition capability and good compatibility with food matrices is an important direction for improving the precise removal of biotoxins.

## 5. Applications of MIPs in Food Contaminant Removal

MIP applications in this field span related but distinct operations. In molecularly imprinted polymer-based solid-phase extraction (MIP-SPE), a contaminant is captured from a small analytical aliquot, matrix components are washed away, and the analyte is often eluted for measurement. A food-decontamination process must instead reduce the contaminant in the bulk edible matrix, recover the sorbent, preserve food quality, and prevent secondary residues. To keep these outcomes separate, this review uses five evidence levels: Level 0, binding in water or synthetic solution; Level 1, analytical cleanup; Level 2, treatment of a spiked or simplified food model; Level 3, edible-matrix decontamination with recovery and quality checks; and Level 4, continuous or pilot validation with throughput, breakthrough, regeneration, economics, and regulatory assessment. Enrichment, sensor response, or spike recovery should not be presented as direct evidence of scalable food decontamination.

### 5.1. Application of MIPs in Plasticizer Removal

For plasticizers, recent architectures improve access to hydrophobic binding sites, but the strongest food-related results remain analytical. A Fe_3_O_4_@MOF@MIP material showed reported capacities of 260 mg g^−1^ for DBP and 240.2 mg g^−1^ for DEHP, reached equilibrium in about 20 min, and gave 70.3–100.7% recovery from drinking water, fruit juice, and white wine over six cycles [79]. These are extraction recoveries from spiked samples, not percentages of contaminant removed from a consumable batch. A 2024 magnetic gallic-acid dummy-template MIP reached 97.43% of its equilibrium DBP uptake within 5 min and was reused for seven cycles [80]. The 97.43% value describes approach to equilibrium and must not be relabeled as solution removal. A MIL-100(Fe)-supported surface MIP reported a Langmuir BPA capacity of 15.45 mg g^−1^, 95% of equilibrium uptake within 10 min, and about 90.1% analytical recovery after 20 cycles [81]. Its tests on food/contact-material extracts likewise used spiking, SPE capture, and solvent elution rather than validated bulk-food treatment.

MIPs can discriminate structurally related phthalates through a combination of cavity shape, hydrogen bonding, aromatic interactions, and hydrophobic partitioning. That advantage is matrix-dependent: in edible oils and dairy products, the target may remain preferentially in the lipid phase, while proteins and lipids can foul exposed surfaces or block pores. Magnetic and surface-imprinted supports shorten diffusion and facilitate separation, but complete particle recovery, metal or MOF release, template leakage, and food-quality retention require direct measurement. Current evidence therefore supports selective analytical extraction and aqueous adsorption more strongly than food-grade plasticizer remediation.

### 5.2. Application of MIPs to Pesticide Residue Removal

The pesticide literature spans both analytical extraction and direct treatment. Davoodi et al. [82] demonstrated selective MIP-SPE of diazinon from aqueous media and cucumber tissue, but the analytical recovery does not by itself establish decontamination. In a more removal-relevant study, a dual-template surface MIP adsorbed 33.01 mg g^−1^ imidacloprid and 25.62 mg g^−1^ acetamiprid and removed 95.8% and 94.8%, respectively, from tea-polyphenol extract while adsorbing only 2.7% of the polyphenols [83]. This coupled contaminant-depletion and product-retention endpoint is stronger than recovery alone, although pilot throughput, sorbent carryover, and long-term regeneration were not established. A 2024 simulation-guided dummy-template MIP removed 90.08–99.04% trifluralin from environmental water, with a capacity of 5.1 mg g^−1^, equilibrium in 5 min, and IF 2.2 [84]. A 2026 dual-template magnetic MIP reported capacities of 27.38 and 19.86 mg g^−1^ for acetamiprid and thiamethoxam and rapid extraction from spiked fruits [85]. The latter remains Level 1 analytical evidence because the analytes were recovered for measurement rather than removed from food intended for consumption.

For pesticide residues, the principal value of MIPs is selective retention in matrices that contain polyphenols, sugars, organic acids, and pigments. Matrix effects in MIP-SPE are reduced rather than eliminated. Selectivity arises from complementary cavity geometry and functional groups, but cleanup also depends on controlled loading, washing, and elution. During loading, the target is retained while weakly bound co-extractives are removed by a selective wash. The target is then eluted by changing solvent composition, pH, or ionic strength [82,83,86].

Proteins, lipids, and highly abundant polyphenols can still occupy nonspecific sites, block pores, alter polymer swelling, or compete through hydrogen bonding and electrostatic interactions. High-fat and protein-rich foods may therefore require dilution, defatting, protein precipitation, or dispersive cleanup before MIP-SPE. Surface-imprinted, porous, and magnetic MIPs shorten diffusion pathways and facilitate sorbent recovery, but they do not replace matrix-specific validation. Studies should report recovery, relative matrix effects, selectivity against a non-imprinted polymer (NIP), template leakage, regeneration, and analyte retention across representative real samples [86]. Dual- or multi-template and dummy-template designs may broaden coverage, although they can introduce site heterogeneity and cross-reactivity. MIPs are therefore selective cleanup media whose performance depends on both cavity design and the complete sample-preparation workflow.

### 5.3. Application of MIPs in Heavy Metal Removal

Jiang et al. [87] applied Pb(II)-imprinted chitosan-coated diatomite to Rhodiola rosea decoction and reported preferential metal removal without a significant change in salidroside, providing an early food-relevant quality endpoint. Recent ion-imprinted studies achieve higher quantitative performance in water. A Pb(II)-imprinted acrylic-acid/acrylonitrile composite had a Langmuir q_max_ of 150.34 mg g^−1^ versus 41.79 mg g^−1^ for the NIP, relative selectivity coefficients of 5.04–9.19 against Ni(II), Zn(II), and Cu(II), and >79% efficiency after eight cycles [88]. A dual-S-site Hg(II)-imprinted MoS_2_/SBA-15 material reported q_max_ = 567.78 mg g^−1^ and 90 min equilibrium [89]. These high-capacity values were obtained in aqueous systems at concentrations and speciation unlike most foods. Before ranking them as food technologies, studies must test competing minerals and chelators, protein binding, pH-dependent speciation, solid recovery, and the preservation of nutritionally desirable elements.

Unlike organic contaminants such as plasticizers and pesticides, the imprinting recognition of heavy metal ions mainly depends on coordination interactions between metal ions and functional monomers or ligands. Therefore, the design of functional monomers, chelating groups, and carrier structures is particularly important. Existing studies have shown that the combination of carriers such as chitosan, diatomite, mesoporous silica, and magnetic nanomaterials with ion imprinting technology can improve the selective adsorption capacity of materials toward metal ions such as Pb^2+^, Cd^2+^, and Cu^2+^. Compared with conventional adsorbents such as ion-exchange resins and activated carbon, ion-imprinted materials are more favorable for the preferential recognition of target ions in systems containing multiple coexisting metal ions, thereby reducing nonspecific effects on mineral elements, proteins, polysaccharides, and bioactive components in foods. In particular, in systems such as traditional Chinese medicine decoctions and plant extracts, the ability to remove heavy metals while maintaining the stability of bioactive components would have high practical value. Nevertheless, current research on heavy metal ion-imprinted materials in the food field remains less extensive than that in environmental water treatment. The main limitations include the risk of material residues, difficulty in post-adsorption separation, insufficient food-grade safety evaluation, and competitive ion adsorption in complex food matrices. Future studies should further develop green carriers, bio-based functional monomers, and magnetically separable structures, while strengthening safety evaluation in food systems to promote the transformation of ion-imprinted materials toward food contaminant removal.

### 5.4. Application of MIPs to Biotoxin Removal

Biotoxin studies also illustrate the difference between recognition and detoxification. Luminescent and hollow-network MIPs have enabled selective enrichment and trace detection of aflatoxin B1 and sterigmatocystin [90,91], but these analytical endpoints do not show that an edible batch is safe after treatment. For patulin in apple juice, Fe_3_O_4_@SiO_2_@CS-GO@MIP provided a capacity of 7.11 mg g^−1^ and removed more than 90% of patulin within 24 h, while magnetic recovery supported reuse [92]. A 2025 dummy-template MIP for four aflatoxins in spiked maize extract gave Langmuir q_max_ values of 7.50–8.38 mg g^−1^, competitive-mixture removal of 70.0–72.6% versus 8.8–11.4% for the NIP, mean IF 6.5, and 77.3–84.5% performance across six cycles [93]. A 3D covalent-organic-framework MIP removed 99.9% microcystin-LR at 1 mg L^−1^ and 99.1% at 6 mg L^−1^ from spiked lake water within 30 min [94]. These water and extract studies demonstrate recognition chemistry, whereas whole-food mass balance, residual sorbent, bioavailability, and post-treatment toxicology remain largely unresolved.

Dummy templates are particularly valuable for highly toxic templates because they reduce target carryover and exposure during synthesis, but leakage of the analogue must still be measured. A 2025 functionalized-graphene MIP removed zearalenone from corn oil while retaining the measured vitamin E and sterol contents [95]. This is a more relevant quality-preservation endpoint than detector recovery, yet the study did not establish pilot throughput, regeneration, particle recovery, extractables, toxicology, or broad sensory equivalence. Surface, porous, and magnetic architectures can improve accessibility and recovery, but their added nanomaterial interfaces increase the need for migration and shedding tests. Biotoxin removal should therefore be judged by residual toxin and toxicity, product quality, complete material balance, and safe adsorbent removal rather than by adsorption capacity alone.

### 5.5. PFAS, Microplastics, and Nanoplastics as Emerging Removal Targets

For PFAS, food-chain relevance is established more strongly than remediation. Recent imprinted resins produced IF values of 7.3 for PFOA and 8.9 for PFOS in egg analysis and IF values of 2.6–5.0 for perfluorocarboxylic acids in milk [62,63]. Both studies were analytical SPE workflows, so their 84.7–104.7% and 75.7–118.1% recoveries must not be interpreted as detoxification of eggs or milk. In synthetic water, a magnetic chitosan MIP reached a PFOA Langmuir q_max_ of 51.28 mg g^−1^, IF 2.65, and >90% capacity retention after five cycles [64]. This result supports electrostatic, hydrogen-bonding, hydrophobic, and cavity-assisted capture, but acidic pH, mg L^−1^ concentrations, and contact times up to 24 h limit direct translation to food or process water. PFAS adsorption also transfers rather than destroys the contaminant, making regenerant treatment and final disposal part of the process.

The gap is wider for microplastics and nanoplastics. A 2026 particle-templated electrochemical MIP recognized 100 and 500 nm polystyrene in water, but it was a sensor and reported no removal capacity [65]. By comparison, non-imprinted full-scale drinking-water processes removed 97.5–100% of particles at least 2 micrometers in ten facilities, while smaller particles and nanoplastics remained outside the measurement scope [66]. Unlike a defined small molecule, a plastic particle varies in polymer chemistry, size, shape, oxidation, additives, aging, biofilm, and protein or lipid corona. Recognition of pristine spherical polystyrene cannot be generalized to aged PE, PP, PET, PVC, fibers, fragments, or nanoplastics in food. Near-term MIP opportunities may therefore lie in selective capture of molecular additives, oligomers, or well-defined particle markers, while whole-particle removal should be benchmarked against filtration, coagulation, flotation, and magnetic separation.

### 5.6. Quantitative Comparison and Evidence Strength of MIP-Based Removal

Table 2 shows why a single performance number cannot establish the superiority of one MIP. Model-derived q_max_, observed uptake, removal percentage, analytical recovery, IF, and residual capacity after reuse describe different outcomes. They are also conditional on initial concentration, sorbent dose, pH, ionic strength, contact time, matrix pretreatment, and whether the target is freely dissolved or bound to food components. The highest capacities in recent metal-ion studies were obtained in synthetic water [88,89], whereas the strongest food-process evidence used a lower-capacity fixed bed but reported 480 L treatment volume, breakthrough, and regeneration [96]. Process relevance therefore increases with evidence level, not necessarily with q_max_.

Across contaminant classes, architecture should be evaluated by the limitation it solves. Surface and porous MIPs shorten diffusion paths; ion-imprinted materials organize coordination ligands; dummy templates reduce target-template carryover; and magnetic supports simplify collection. Each strategy also introduces a trade-off: surface layers can foul, ion selectivity changes with speciation and chelators, dummy templates can cross-react or leak, multi-template sites compete, and magnetic or MOF components add a particle- or metal-release question. A credible comparison therefore requires matched NIP and bare-support controls, competitive mixtures, dynamic capacity, recovery completeness, contaminant mass balance, and retained food quality.

MIPs should not be presented as a universal substitute for washing, activated carbon, resins, membranes, oxidation, or biodegradation. Their strongest potential is in narrowly defined separations where conventional treatment also removes valuable food constituents or cannot discriminate a target from close analogues. At present, direct food evidence is concentrated in tea-polyphenol extracts, apple juice, corn oil, and wine, while many newer high-capacity studies remain at Levels 0–1. Hybrid processes may be more realistic: conventional operations reduce bulk contaminant load, and a recoverable MIP polishing step provides selective final control.

## 6. Challenges Facing MIPs

The transition from laboratory recognition to food processing is constrained by manufacturing, matrix, safety, and process requirements. Batch reproducibility should be demonstrated across at least three independent production lots using predefined specifications for particle size, porosity, residual template and monomers, accessible capacity, selectivity, mechanical strength, and magnetic or coating stability. A high-performing milligram-scale batch is insufficient when kg-scale synthesis changes heat transfer, mixing, nucleation, washing efficiency, and site heterogeneity [97].

The consistency of batch preparation and structural controllability of MIPs remain important factors limiting their wider application. Wackerlig [98] investigated nanoscale MIPs and found that nano-MIPs possess high specific surface area and good chemical stability. However, commonly used methods such as precipitation polymerization and emulsion polymerization still suffer from limited yield, uneven particle size distribution, and complex processing procedures. For the removal of food contaminants, material particle size, pore structure, recognition site distribution, and surface properties can all affect adsorption rate, selectivity, and regeneration performance. Therefore, achieving high-throughput, low-cost, and reproducible preparation of MIPs remains a key issue.

In addition, selective recognition and safety evaluation in complex food matrices remain insufficient. Food systems often contain various coexisting components, such as proteins, lipids, polysaccharides, polyphenols, pigments, and inorganic salts, which may compete with target contaminants for adsorption sites and reduce the recognition efficiency of MIPs. Meanwhile, template residue, residual polymeric monomers or crosslinkers, migration of nanomaterials, and incomplete separation of adsorbents may also introduce risks of secondary contamination. Therefore, the application of MIPs in real food systems requires not only the evaluation of adsorption capacity and selectivity, but also systematic investigation of their effects on food nutrition, flavor, bioactive components, and processing quality.

Commercial and regulatory progress is best described as early and product-specific. A 2025 semi-commercial study treated 480 L of wine per replicate with approximately 0.7–1.0 kg of a commercial MIP fixed bed, removed up to 47% of free volatile phenols, and observed breakthrough after about 28–80 bed volumes [96]. Effective regeneration required concentrated ethanol, glycoconjugated precursors largely passed through, sensory benefit was matrix-dependent, and only one resin batch was evaluated. The same cross-linked polyester wine-resin use received a 2024 administrative approval from the U.S. Alcohol and Tobacco Tax and Trade Bureau and a 2026 FSANZ product-specific assessment [99,100]. These decisions do not constitute class-wide approval of methacrylate powders, magnetic nanoparticles, graphene composites, MOF-supported MIPs, other contaminants, or other foods. Regulatory readiness must be established for the exact formulation, particle form, process, jurisdiction, and conditions of use.

For direct food contact, insolubility is not an adequate safety argument. Validation must include targeted and non-targeted extractables/leachables, template and dummy-template migration, residual monomer/crosslinker/initiator/porogen, nanoparticle or metal release, complete sorbent recovery, and carryover after regeneration. Continuous processing additionally requires pressure-drop and breakthrough models, clean-in-place compatibility, microbial control, hundreds-of-cycle lifetime data, and retirement criteria. Techno-economic and life-cycle comparisons should report resin cost, energy, solvent and water use, food yield loss, downtime, waste toxicity, and cost per treated mass or contaminant removed. Without these data, low cost, sustainability, food-grade status, and industrial readiness remain development goals rather than demonstrated advantages.

## 7. Future Development Directions for MIPs

The future development of MIPs in the field of food contaminant removal should focus on green preparation, precision, intelligence, and practical application. First, in terms of material preparation, the selection of template molecules, functional monomers, crosslinkers, and solvent systems should be further optimized to improve the selectivity, stability, and adsorption performance of MIPs. Previous studies have indicated that optimizing the molecular structures and physical properties of MIPs can enhance their adaptability and effectiveness under different environmental conditions. In the future, the use of toxic organic solvents and high-risk template molecules should be reduced, and strategies such as aqueous-phase polymerization, bio-based monomers, green crosslinkers, dummy templates, and degradable carriers should be developed to improve the environmental friendliness and food safety of MIPs. Meanwhile, complete template elution and control of template leakage remain critical issues in the practical application of MIPs. Therefore, future research should strengthen the optimization of elution methods and the evaluation of material residues to avoid the risk of secondary contamination. Ali [101] emphasized the importance of optimizing MIP preparation methods and properties. By improving molecular structures and physical characteristics, the adaptability and effectiveness of MIPs under various environmental conditions can be enhanced. Future research directions include improving MIP performance and stability, developing novel synthesis techniques and surface modification strategies, and exploring the application of nanotechnology.

In terms of functional monomer and recognition site design, the matching between the structural characteristics of target contaminants and the interaction mechanisms of monomers should be strengthened. Functional monomers form stable complexes with template molecules through hydrogen bonding, electrostatic interactions, hydrophobic interactions, π–π interactions, or coordination interactions, and are therefore key factors determining the recognition ability and selectivity of MIPs. Related studies have shown that optimization of functional monomer structures and the introduction of composite carriers such as porous conductive materials can improve the recognition stability and detection sensitivity of molecular imprinting systems [102]. Therefore, for different contaminants such as plasticizers, pesticide residues, heavy metals, and biotoxins, targeted functional sites and pore structures should be constructed according to their molecular structures, polarity, hydrophilicity or hydrophobicity, and binding mechanisms.

In terms of material structure regulation, surface imprinting, magnetic separation, core–shell structures, mesoporous structures, and nanocomposites should be employed to improve the accessibility of recognition sites and mass transfer efficiency. Conventional MIPs often suffer from buried binding sites, slow adsorption kinetics, and insufficient compatibility with complex matrices. Surface imprinting and porous carriers can shorten the diffusion pathways of target contaminants, while magnetic materials facilitate the rapid separation of adsorbents from food systems. In addition, dual-template, multi-template, and multi-step recognition strategies can improve the ability of MIPs to distinguish structurally similar contaminants and enhance their selectivity in complex matrices [103].

Rational design should move beyond increasing batch capacity. Molecular simulation and data-driven screening can prioritize monomers, solvent compatibility, and dummy templates, while surface, membrane, monolith, and fixed-bed formats can improve accessible dynamic capacity and recovery. For PFAS, studies should include short- and ultrashort-chain compounds, precursors, realistic mixtures, ng L^−1^ or ng kg^−1^ concentrations, natural organic matter, and regenerant disposal. For microplastics and nanoplastics, validation should cover aged PE, PP, PET, PS, and PVC particles, fragments and fibers, micro- and nanoscale size distributions, and realistic protein, lipid, or biofilm coronas. Stimuli-responsive systems may facilitate desorption, but added functionality must be weighed against leachables, process complexity, and regulatory burden.

A staged translation pathway is needed. First, studies should compare MIP, NIP, bare support, and an established treatment under identical matrix and concentration conditions. Second, whole-food experiments should report mass balance, naturally contaminated or realistic spikes, sorbent recovery, migration, nutrients, color, flavor, texture, and residual toxicity. Third, flow-through trials should report dynamic capacity, breakthrough, pressure drop, cleaning, regeneration, carryover, and performance across lots. Finally, pilot studies should add HACCP integration, worker and consumer safety, waste handling, techno-economics, life-cycle impacts, and jurisdiction-specific authorization. This progression would convert molecular selectivity from an attractive laboratory property into a defensible food-processing function.

## 8. Conclusions

This review evaluates molecular imprinting as a selective separation strategy for plasticizers, pesticide residues, heavy metals, biotoxins, and emerging contaminants. Its principal conclusion is that analytical recognition, aqueous adsorption, and food decontamination are not interchangeable evidence. Recent MIPs can show rapid uptake, high model capacities, useful IF values, and reuse, but many results remain based on synthetic water or spiked extracts. Stronger food evidence includes pesticide removal from tea-polyphenol extracts, patulin adsorption from apple juice, zearalenone treatment of corn oil, and semi-commercial fixed-bed removal of smoke-taint phenols from wine. Even these studies leave gaps in multi-lot reproducibility, extractables and leachables, complete sorbent recovery, long-term regeneration, sensory and nutritional equivalence, economics, and regulatory portability. MIPs should therefore be regarded as promising, selectively engineered sorbents and polishing media rather than an already validated universal replacement for conventional removal technologies. Future progress will depend less on another high batch q_max_ and more on dynamic, matrix-matched, food-safe, and independently scalable process evidence.

## Figures and Tables

**Table 1:** Types, Sources, and Characteristics of Major Chemical Contaminants in Food [58,59,60].

Contaminant Category	Representative Compounds	Major Sources	Key Characteristics	Health Risks	Removal Challenges
Plasticizers	Dibutyl phthalate (DBP), di(2-ethylhexyl) phthalate (DEHP), and other phthalate esters (PAEs)	Food packaging materials, processing equipment, illegal addition	Strong lipophilicity, high migration potential, and good stability	Endocrine disruption, reproductive toxicity, and liver damage	Prone to accumulation in lipid-rich foods; conventional adsorbents show poor selectivity
Pesticide residues	Organophosphorus pesticides, organochlorine pesticides, and carbamates	Agricultural pesticide application and environmental contamination	Persistence and structural diversity	Neurotoxicity, carcinogenicity, and mutagenicity	Low residue concentrations and combined contamination make complete removal difficult
Heavy metals	Pb, Cd, Hg, and As	Soil, water, industrial emissions, and environmental migration	Non-degradability and bioaccumulation	Kidney injury, nervous system damage, and carcinogenicity	Difficult to separate and often coexist with nutritional components
Biotoxins	Aflatoxins, shellfish toxins, and bacterial exotoxins	Fungal contamination, algal toxins, and bacterial contamination	High thermal stability and strong toxicity	Hepatotoxicity, immunosuppression, and acute poisoning	Difficult to degrade by heat treatment; conventional processes are insufficient for effective removal
Emerging contaminants [61,62,63,64,65,66]	PFAS, microplastics, and nanoparticles	Packaging materials and environmental exposure	Recalcitrance, structural stability, and persistent contamination	Metabolic disruption and potential carcinogenicity	Lack of standardized removal methods and complex enrichment mechanisms

**Table 2:** Quantitative comparison of representative MIP studies and their evidence level. Values are reported as stated by the original studies and are not directly rankable across different concentrations, phase ratios, matrices, and model definitions.

Target and Matrix	MIP Architecture	Evidence Level	Capacity and Removal/Recovery	Kinetics, Selectivity, and Reuse	Critical Limitation and Reference
Imidacloprid and acetamiprid; tea-polyphenol extract	Dual-template surface MIP on silica	Level 2: model-food treatment	q_max_ 33.01 and 25.62 mg g^−1^; removal 95.8% and 94.8%; tea-polyphenol adsorption 2.7%	Langmuir behavior; MIP > NIP; reuse reported	Direct depletion with a quality-retention indicator [83]
Patulin; apple juice	Fe_3_O_4_@SiO_2_@CS-GO surface MIP	Level 2: food-matrix batch treatment	Capacity 7.11 mg g^−1^; >90% removal within 24 h	Magnetic recovery; reusable; cycle-resolved capacity not reported	Long contact time and incomplete process/safety validation [92]
DBP/DEHP; water, juice, and white wine	Fe_3_O_4_@MOF@MIP-160	Level 1: analytical cleanup	Reported capacities 260 and 240.2 mg g^−1^; recovery 70.3–100.7%	Equilibrium about 20 min; stable across six cycles	Recovery after extraction is not bulk-food decontamination [79]
DBP; water	Magnetic gallic-acid dummy-template MIP	Level 0: proof of binding	97.43% of equilibrium uptake reached in 5 min; q_max_ and removal percentage not reported	Pseudo-second-order/Freundlich fits; seven reuse cycles	Kinetic fraction must not be read as removal efficiency [80]
BPA; food/contact-material extracts	MIL-100(Fe)-supported surface MIP	Level 1: analytical cleanup	Langmuir q_max_ 15.45 mg g^−1^; recovery 80.67–100.58%	95% of equilibrium uptake in 10 min; about 90.1% recovery after 20 cycles	Spiked SPE extracts, not treated food for consumption [81]
Trifluralin; environmental water	Simulation-guided dummy-template MIP	Level 0: proof of binding	Capacity 5.1 mg g^−1^; removal 90.08–99.04%	Equilibrium 5 min; IF 2.2; selectivity factor 1.2–3.0	Food-matrix and continuous-flow evidence absent [84]
Pb(II); deionized water	Acrylic-acid/acrylonitrile ion-imprinted composite	Level 0: proof of binding	Langmuir q_max_ 150.34 mg g^−1^ versus 41.79 mg g^−1^ for NIP	Equilibrium 105 min; relative selectivity 5.04–9.19; cycle 8 efficiency > 79%	High-loading synthetic water; food speciation not tested [88]
Hg(II); water	Dual-S-site MoS_2_/SBA-15 ion-imprinted polymer	Level 0: proof of binding	Langmuir q_max_ 567.78 mg g^−1^; analytical recovery 97.92–100.28%	Equilibrium 90 min; qualitative co-ion selectivity	Recovery is not a removal endpoint; regeneration details limited [89]
Microcystin-LR; spiked lake water	3D COF cavity MIP; L-arginine pseudo-template	Level 0: food-relevant water	99.9% removal at 1 mg L^−1^ and 99.1% at 6 mg L^−1^	30 min treatment; toxin q_max_ and reuse not reported	Strong water evidence but not food-matrix detoxification [94]
Four aflatoxins; spiked maize extract	5,7-Dimethoxycoumarin dummy-template MIP	Level 1: analytical cleanup	Langmuir q_max_ 7.50–8.38 mg g^−1^; competitive removal 70.0–72.6% versus 8.8–11.4% for NIP	Equilibrium 15 min; mean IF 6.5; six cycles retained 77.3–84.5%	Spiked extract, without whole-food quality or toxicology endpoints [93]
Smoke-taint volatile phenols; wine	Commercial cross-linked polyester MIP fixed bed	Level 4: semi-commercial process	480 L per replicate; up to 47% free-phenol removal	Breakthrough at about 28–80 bed volumes; 96% ethanol enabled regeneration	Glycoconjugates largely passed through; one resin batch [96]
PFOA; synthetic water	Magnetic chitosan/MAA MIP	Level 0: proof of binding	Langmuir q_max_ 51.28 mg g^−1^; IF 2.65	>90% capacity retained after five cycles; magnetic separation in 30 s; contact up to 24 h	Acidic, mg L^−1^ batch conditions; no food, column, or leaching test [64]

## Data Availability

No new data were created or analyzed in this study. Data sharing is not applicable to this article.
